# Phytochemical Fingerprinting and Activity of Extracts from the Leaves of *Dolichos kilimandscharicus* (Fabaceae) on Jurkat-T Cells

**DOI:** 10.1155/2020/1263702

**Published:** 2020-10-05

**Authors:** Simbarashe Sithole, Paul Mushonga, Llewellyn N. R. Nhamo, Godloves Fru Chi, Stanley Mukanganyama

**Affiliations:** ^1^Department of Biochemistry, University of Zimbabwe, Mt. Pleasant, Harare, Zimbabwe; ^2^Department of Chemistry, University of Zimbabwe, Mt. Pleasant, Harare, Zimbabwe; ^3^Department of Organic Chemistry, University of Yaoundé 1, Cameroon

## Abstract

Plants are a source of over a quarter of the prescription drugs currently in use worldwide. Zimbabwe has a rich plant biodiversity with only a limited number reported for the treatment of cancer. The leaf extracts of *Dolichos kilimandscharicus* were selected for the screening of their antiproliferative efficacy and cytotoxicity effects. This plant has increasingly been used by local folk as a treatment for cancer or cancer-related symptoms though its bioactivity has not been scientifically determined. This investigation also sought to identify constituent compounds in the crude extract preparations responsible for their antiproliferative efficacy. The antiproliferative effects of six-leaf extracts on Jurkat-T *in vitro* were investigated using the Trypan blue exclusion assay. The extracts were tested with increasing concentration, using chlorambucil as a standard anticancer drug. Cytotoxicity of extracts was determined against RAW 264.7 cells using a colorimetric tetrazolium-based assay. In additionthe ability of the extracts to induce apoptosis was determined for the most potent leaf extracts. The order of potency of the leaf extracts of *D. kilimandscharicus* against Jurkat-T cell line was found to be MeOH < Ethyl Acetate < DCM: MeOH < EtOH with IC_50_s of 33.56, 30.44, 22.93, and 21.59 *μ*g/mL, respectively. Furthermore, the most potent extracts exhibited very low cytotoxicity against all the tested cells. *D. kilimandscharicus* leaf extracts induced apoptosis in the Jurkat-T cells as was shown by DNA fragmentation. UPLC-MS analysis of crude extracts led to the identification of 23 compounds from the ethanol extract and these may be responsible for the observed antiproliferative effects. Rutin, quercetin, luteolin, apigenin, hispidulin, kaempferol derivatives, as well as caffeoylquinic acid are some of the compounds identified in the extracts. The results of this study showed that the ethanol and ethyl acetate leaf extracts of *D. kilimandscharicus* have antiproliferative activity against Jurkat-T cells and may act by inducing apoptosis.. The current findings offer supporting evidence for the use of these plant species in the treatment of cancer in ethnomedicinal practices.

## 1. Introduction

The World Health Organization estimated cancer deaths to be over 9.6 million in 2018, with most of these deaths in low- and medium-income countries [1]. The cost of cancer treatment is beyond the reach of most cancer patients in Zimbabwe. Though access to medical care is generally poor, the situation for cancer patients is worse due to minimal health facilities offering cancer treatment. Leukaemia is a cancer of the bone marrow and blood, marked by distorted proliferation and development of leukocytes and their precursors in the blood and bone marrow. It is estimated that in Zimbabwe, 17 465 cancer cases were diagnosed in 2018 alone [1]. Leukemic cell lines represent an essential instrument for the biological characterization of human leukaemia. Jurkat-T cells are an IL-2 producing T lymphocyte cell line, shown to express T cell characteristics and complement receptors [2]. Jurkat-T cells are easy to culture, and grow rapidly, and can be used in human immunodeficiency virus research, though they are contaminated by previously undetected retroviruses [3].

A major part of traditional therapy involves the use of plant extracts or their active principles to treat ailments [4]. Natural products may be sources of compounds that are lead compounds for application in various pharmacological, physiological, and biochemical studies [5]. Several studies on medicinal plants have highlighted the dependence of a large percentage of the world population on traditional medicine for the management of an array of ailments [6]. These ethnobotanical surveys on plants traditionally show that these medicinal plants are used either alone as a primary therapeutic choice or in conjunction with conventional medicines [7]. Though there is this dependence on herbal medicines, often, clinicians are either unaware of their patients' use of herbs as well as the specific herb being used. Traditional healers further complicate matters, as they are unwilling to divulge the identity of their herbal concoctions that they consider “safe.” This makes it difficult to ascertain if the herb may be a contributor to the healing properties or failure of treatment. WHO Traditional Medicine Strategy, in realising the increasing importance of traditional medicine in various healthcare systems around the world, has recently set out to promote the safe and effective use of traditional medicine [8]. There is, however, a need to carry out systematic screening of these plants to validate their use in folk medicine and to reveal the active compounds by isolation and characterisation of their constituents.

Advanced biological experimental procedures, which include cell viability and cytotoxicity assays, are essential for drug screening in determining the safety of drug molecules and herbal products [9] in vitro. There is a need to acquire knowledge of the toxicological profile of the plants used traditionally to anticipate the ingestion of toxic plants. The 3-(4,5-dimethylthiazol-2-yl)-2,5-diphenyltetrazolium bromide (MTT) assay is the more commonly employed assay for cytotoxicity screening of bioactive compounds which measure for cell death, inhibition of cell growth, or cell proliferation [10] in vitro. Probable risks associated with herbal medicine use have been reported and are of major concern in herbal medicine toxicity [11].

Compounds from herbal medicines have become the default treatment option used in chemotherapy as they have a variety of different structures and mechanisms of action [12]. Apoptosis, also termed programmed cell death, is a gene-directed form of cell death that is evident typically in cell morphology. Generally, mammalian cells undergo apoptosis during normal development or as a result of a variety of stimuli, which are not limited to DNA damage, growth factor deprivation, and abnormal expression of oncogene or tumour suppressor genes [13–15]. Apoptosis is widely viewed as an important mechanism in the reduction of cell growth. Inhibition of the enzymes in the pathway renders most of the chemotherapeutic drugs ineffective. In a study by Raskin et al. [12], it was shown that plants possess diverse compounds with multiple therapeutic effects which can be used to counter the inhibition of enzymes to reduce the ineffectiveness of drug.

*Dolichos kilimandscharicus subsp. kilimandscharicus* has been used as a natural fungicide in small-scale farming, as a nonvertebrate poison mainly applied in fishing [16]. The root slurry of *D. kilimandscharicus* is used in Ethiopia to treat sorghum smuts, and the methanolic crude extracts from *D. kilimandscharicus* were shown to possess *in vitro* antifungal activity with *Botrytis cinerea* [17]. A few ethnomedicinal uses have been documented on the plant. The roots are used for the treatment of aching limbs [18], while in Kenya, Uganda, and Tanzania, the leaves are boiled together with other plants for the treatment of colic, dysentery, gonorrhoea, and syphilis [19]. There are few research reports on the phytochemical profiles of *D. kilimandscharicus*. As part of an investigation of the antiproliferative properties as well as the composition of the leaf extracts of *D. kilimandscharicus* seven extracts prepared from the plant were investigated to determine if they posessed antiproliferative properties against cancer cells in vitro.

This study was aimed at determining the antiproliferative, cytotoxic effects on Jurkat-T cells as well as determine the profile of possible phytocompounds of the leaf extracts of *D. kilimandscharicus*. Further to this, the study also sought to explore whether the leaf extracts induced apoptosis as a biochemical mode of action for the oberserved effects.

## 2. Materials and Methods

### 2.1. Chemicals

All chemicals, sera, media, and drugs used were purchased from Sigma-Aldrich (Steinheim, Germany) and were of analytical grade. These included foetal bovine serum (FBS), Roswell Park Memorial Institute 1640 media (RPMI), reduced L-glutathione (GSH), penicillin, neomycin, and streptomycin solution (PNS), Hanks Buffered Saline Solution (HBSS), acetone, dimethyl sulfoxide (DMSO), agarose, ethidium bromide (EtBr), Trypan blue dye, monochlorobimane (MCB), 2,4-dinitrochlorobenzene, and chlorambucil.

### 2.2. Plant Collection and Extract Preparation

D. kilimandscharicus was collected from Centenary (17°S, 31°E) Zimbabwe. Mr. Christopher Chapano, a taxonomist at the National Botanic and Herbarium Gardens (Harare, Zimbabwe), authenticated the plant. Herbarium samples were kept at the Department of Biochemistry, University of Zimbabwe (DK2017C). The leaves were air-dried in an oven at 40°C and ground to a powder using a pestle and mortar. Serial exhaustive extraction was done with a sample to solvent ratio of 1 : 5. The solvents used for serial exhaustive extraction were in the order of increasing polarity starting with hexane, dichloromethane, ethyl acetate, acetone, ethanol, methanol, and ending with water. The extracts were filtered and evaporated under vacuum until a constant dry weight of each extract was obtained and the residues stored at 4°C until required. The extract solutions for testing were prepared by dissolving the plant extract in DMSO and RPMI media, with a final concentration of DMSO when added to test well 0.83%.

### 2.3. UPLC-MS Analysis of Compounds

Phytofingerprinting was performed on the extracts using a Waters Acquity UPLC system (Waters Corporation, Milford, MA, USA) with an Acquity BEH C18 column (2.1 mm × 100 mm, 1.7 *μ*m particle size) incorporating a binary pump, vacuum degasser, autosampler, column oven, and Micromass Xevo tandem quadrupole mass spectrometric detector (QTOF Xevo G2; Waters Micromass, Manchester, UK) equipped with ESI probe. Gradient elution was performed at a flow rate of 0.1 mL/min throughout at injection volumes of 10 *μ*L. Gradient parameters were adjusted by systematically changing the percentage of organic modifier at initial conditions, and/or the isocratic hold period at initial conditions, and/or gradient steepness. Electrospray mass spectra data were recorded on a negative ionization mode for a mass range *m*/*z* 100 to *m*/*z* 1500 at a collision energy of 50 V. Relative quantification of some constituents of the extracts was performed. Possible compounds that may be found in the extract were determined using fragmentation data and comparing it to a series of publications on UPLC-MS.

### 2.4. Determination of the Effects of Leaf Extracts on Jurkat-T Cells

The Jurkat E6.1 Human Leukemic T Cell Lymphocytes cell line obtained from Sigma-Aldrich, (ECACC CAMR Salisbury Wiltshire, UK) were maintained in a fully humidified atmosphere containing 5% CO_2_, at 37°C at a density of approximately 1 × 10^5^ cells per mL in a Shel lab incubator (Sheldon Mfg. Inc,Cornelius, USA). RPMI-1640 medium, supplemented with 10% heat-inactivated fetal bovine serum (FBS) and 1% antibiotics (penicillin 5000 U, streptomycin 5 mg/mL, neomycin 10 mg/mL) was the media used for maintaining the cells. Calculation of cell concentration as well as the assessment of cell viability via Trypan blue staining was done by counting cells under a microscope using a haemocytometer. The effect of the seven leaf extracts were screened at 100 *μ*g/mL (final concentration in the wells), with chlorambucil used as a positive standard, on Jurkat-T cells with the volume of cells containing 1 × 10^5^ cells/mL. Extracts that reduced the growth of two of the cell lines to 75% or less were subjected to further *in vitro* investigation for the dose-dependent effect at four concentrations. Results were reported as IC_50_ (50% inhibitory concentration) calculated for each extract. Cells that served as negative controls were exposed to 0.1% DMSO only. The cells were maintained in an incubator at 37°C in a humidified atmosphere with 5% CO_2_. Cell counts were done after 72 hrs using the Trypan blue dye exclusion assay. DNA fragmentation of the most potent leaf extracts at four concentrations was carried out to investigate for the induction of apoptosis.

### 2.5. Determination of the Induction of Apoptosis by the Extracts

Jurkat-T cells were treated with leaf extracts of *D. kilimandscharicus* at 0, 31.25, 62.5, 125, and 250 *μ*g/mL (final concentration in the wells) for 72 hrs before being used for this experiment. Cells exposed to chlorambucil at 16 *μ*g/mL, and the vehicle control (DMSO) only were used as the positive and negative controls, respectively. Briefly, cells were centrifuged at 12 000 rpm for 5 min in a KK Centrifuge (Gemmy Industrial Corp., Taiwan) and then washed with PBS (pH 7.2). After discarding the supernatant, 200 *μ*L of lysis solution (10 mM Tris (pH 7.4), 5 mM EDTA, 0.2% Triton X-100), and 10 *μ*l of 1 mg/mL proteinase K were added. The cells were left in a 56°C water bath overnight (Shaker Bath SBS30, Stuart Scientific, UK). Eight microliters of RNase (100 *μ*g/mL) was added to each tube, and then the incubation continued at 37°C for 1 hr. A 20 *μ*L volume of 1.5 M NaCl was added, and then the tubes were inverted several times before being centrifuged at 12 000 rpm for 15 min. The supernatant was added to clean tubes. Ice-cold isopropyl alcohol (2 x the volume) was added. The tubes were inverted several times and left at -80°C for 1 hr. The tubes were centrifuged at 12 000 rpm for 15 min in a microcentrifuge (Centrifuge 5415C, Eppendorf, Berlin, Germany), and the supernatant was then discarded. The isolated DNA was allowed to air-dry before being resuspended in TE buffer (10 mM Tris-HCl (pH 7.4) and 0.5 mM EDTA). The pattern of DNA cleavage was analyzed by agarose gel electrophoresis using a Bio-Rad electrophoresis unit (Bio-Rad, Hercules, USA). DNA was stained with ethidium bromide.

### 2.6. Cytotoxicity Studies of the Leaf Extracts of *D. kilimandscharicus*

RAW 264.7 cells obtained from Sigma-Aldrich, (ECACC CAMR Salisbury Wiltshire, UK) were grown and maintained in T-25 flasks (Corning Incorporated, USA) using complete Dulbecco's Modified Eagle's Medium (DMEM). Cells were kept at 37°C for 72 hrs in a 5% CO_2_ incubator (Sheldon Mfg. Inc, Cornelius, USA) and observed daily. At approximately 90% confluence, they were trypsinized and concentrated at 1 800 rpm for 5 min. The pellet was resuspended and diluted to get 1 × 10^4^ cells per well. Cells were then seeded (100 *μ*L) in 96-well cell culture plates (Costar, USA) followed by incubation overnight to allow cell adhesion. A 10 *μ*L volume of serially diluted compound solutions were added in duplicate after 24 hrs of seeding then incubated for 72 hrs in a humidified atmosphere at 37°C and 5% CO_2_. The ethyl acetate and ethanol fractions were screened at variable concentrations (1000, 200, 40, 8, and 1.6 *μ*g/mL). DMSO was added as a negative inhibitor at 10% (*v*/*v*), while the positive control was chlorambucil. A 10 *μ*L volume of resazurin (0.15 mg/mL in PBS) stock solution was added to each well, gently mixed, and incubated for an additional 4 hrs. Fluorescence was measured using Magellan Infinite M200 fluorescence multiwell plate reader (Tecan) at an excitation and an emission wavelength of 530 and 590 nm, respectively. Dose-response curves were constructed to determine the median cytotoxic concentration (CC_50_) by using GraphPad™ version 6 for Windows.

### 2.7. Statistical Analysis

Data were analysed using GraphPad™ version 6 for Windows (GraphPad™ Software Inc., San Diego, California, USA). All data were expressed as mean ± standard deviation of the mean. Statistically significant differences between the mean of the controls and the tests were analysed using one-way ANOVA with Dunnett's Multiple Comparison Post Test with *P* value being 0.05.

## 3. Results and Discussion

### 3.1. Antiproliferative Effects of Leaf Extracts on Jurkat-T Cells

**Figure 1:**
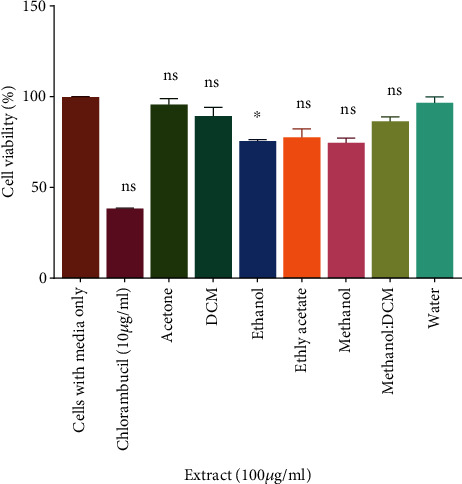
Antiproliferative effects of the extracts of *D. kilimandscharicus* against Jurkat-T cells: Exponentially growing Jurkat-T cells at a concentration of 1.0 × 10^5^ cells/mL were seeded in 24–well culture plates with various leaf extracts of *D. kilimandscharicus* at a concentration of 100 *μ*g/mL in a volume of 100 *μ*L. The plates were incubated at 37°C for 72 hrs, viable cells density being counted by Trypan blue exclusion in a haemocytometer. The differences between cells with media only and treated cells were determined by the *t*-test, and the significance threshold was set to *P* ≤ 0.05. (^ns^*P* > 0.05, ^∗^*P* < 0.05 vs. cells with media only).

The activity of the leaf extracts of *D. kilimandscharicus* on the proliferative activity of Jurkat-T cells was evaluated using the MTT assay. The criteria we used for determining the antiproliferative, as well as cytotoxic effects of crude extracts, has been established by the United States National Cancer Institute, which stipulates that an extract may be considered promising when searching for activity if the IC_50_ is less than 30 *μ*g/mL in a screening assay [20]. The Jurkat-T cancer cell line was exposed toseven leaf extracts of *D. kilimandscharicus* at a concentration of 100 *μ*g/mL and incubated for 72 hrs. The ethanol, ethyl acetate, methanol, and methanol: DCM leaf extracts of this plant were shown to possess phytochemicals that inhibited cell growth of Jurkat-T cells *in vitro*. The high concentrations (100 *μ*g/mL) of the ethanol leaf extract were able to significantly (*P* ≤ 0.05) inhibit cell growth of the cancer cell line when compared with control (Figure 1). In contrast, at the same concentrations, the DCM, acetone, and water leaf extracts had no toxic effect on Jurkat-T cells. In the initial tests, 100 *μ*g/mL of each extract was tested against Jurkat-T cell line, and, from the results, the active extracts considered were those which gave less than 75% survival at exposure time 72 hrs.

The results from this study showed that leaf extracts of *Dolichos kilimandscharicus* exhibited high antiproliferative activity, with the ethanol and ethyl acetate being more effective against Jurkat-T cells. The water extract, however, showed no activity against the cancer cell line at the highest extract concentration. Calculation of the IC_50_ values for the leaf extracts of the two most potent leaf extracts (ethanol–22 *μ*g/mL and ethyl acetate–30 *μ*g/mL) confirmed that the ethanol extract of *D. kilimandscharicus* was more effective against Jurkat-T cells but with less activity on the normal cells (422 *μ*g/mL).

**Figure 2:**
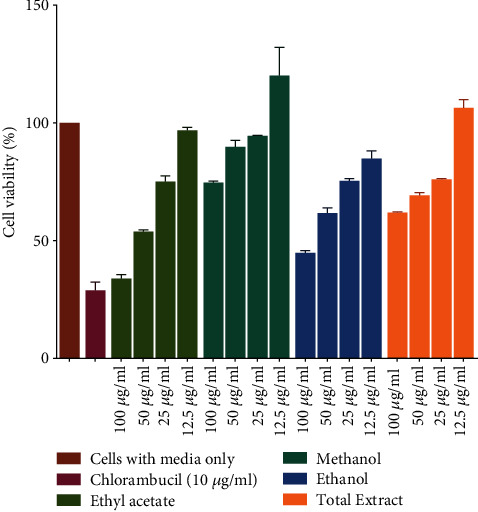
Inhibition activity of various concentrations of four most potent leaf extracts (ethyl acetate, methanol, ethanol, and methanol: DCM) on cellular growth of Jurkat-T cells at different extract concentration (12.5, 25, 50, and 100 *μ*g/mL). The plates were incubated at 37°C for 72 hrs and cell growth was then analysed using the MTT assay. Three replicate values were used to determine the cytotoxicity of each extract.

The 3-(4, 5-dimethylthiazol-2-yl)-2, 5-diphenyltetrazolium bromide (MTT) assay was used for the investigation of the antiproliferative action of the four most potent leaf extracts (Figure 2). The active extracts were diluted in medium to produce 4 concentrations of 12.5, 25, 50, and 100 *μ*g/mL of each extract. The *D. kilimandscharicus* leaf ethanol extract (IC_50_ = 21.59 *μ*g/mL) was the most active against Jurkat-T cells at exposure time 72 hours, while the ethyl acetate extract of *D. kilimandscharicus* leaf was the second most effective (IC_50_ = 30.44 *μ*g/mL).

### 3.2. Effects of Plant Extracts on the Induction of Apoptosis in Jurkat-T Cells

**Figure 3:**
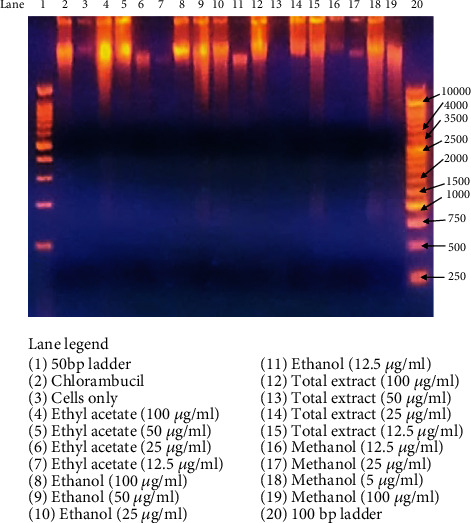
Agarose gel electrophoresis demonstrating DNA fragmentation. Jurkat-T cells were treated with ethyl acetate, ethanol, methanol, and methanol: DCM extracts at concentrations of 100–12.5 *μ*g/mL for 72 hrs and then gel electrophoresis was run to investigate the induction of DNA fragmentation in a dose-dependent manner. The gel was observed under UV light (UVP–Benchtop Variable Transilluminator, M-26V, Upland, CA, USA). Chlorambucil in lane 2 was used as the positive control at a concentration of 10 *μ*g/mL; Jurkat-T cells were incubated in RPMI media only in lane 3 as the negative control.

Apoptosis was investigated by measuring the percentage of fragmented DNA. To elucidate whether the leaf extracts of *D. kilimandscharicus* inhibited Jurkat-T cell proliferation through induction of apoptosis, we examined the cell death by DNA fragmentation after 72 hours (Figure 3). DNA fragmentation(Figure 3)showed that for all four extracts, a concentration of at least 50 *μ*g/mL of extract was optimum enough to induce fragmentation in Jurkat-T cells. The results demonstrated a typical DNA “ladder” pattern in Jurkat-T cells treated with 10 *μ*g/mL of chlorambucil, the positive control. This feature was also evident in cells exposed to the leaf extracts. Several agents capable of inducing apoptosis as well as inhibiting cell proliferation are currently being used for the treatment of cancer [15].

Several agents capable of inducing apoptosis as well as inhibiting cell proliferation are currently used for the treatment of cancer. DNA fragmentation assay was carried out according to the method of Moyo et al. [21] with various concentrations of four most potent extracts. It was found that the ethanol,ethyl acetate, DCM: methanol and methanol leaf extracts of *D. kilimandscharicus* induced DNA fragmentation within 72 h. ( DNA fragmentation was observed in cells treated with leaf extracts between 12.5 and 100 *μ*g/mL concentrations. A detailed study into the mechanisms responsible for the induction of apoptotic cell death must further be elucidated to ascertain the antiproliferative effect of the *D. kilimandscharicus*. Previous studies by Mitsuhashi et al. [22] on the molecular mechanism underlying the effect of a triterpenoid isolated from the plant *Zizyphus jujube* showed that plant-derived compounds may lead to apoptotic cell death in human leukaemic cells.

### 3.3. Cytotoxicity Effects of Leaf Extracts *In Vitro*

**Figure 4:**
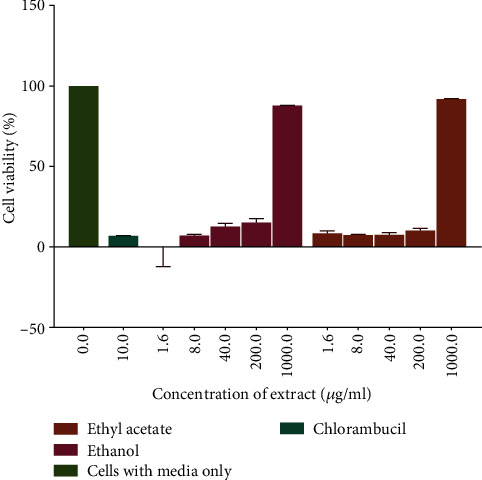
The cytotoxicity of RAW 264.7 cells induced by exposure to the ethanol and ethyl acetate leaf extracts. A plot of percentage cell viability versus extract concentration was used to determine the concentration of the extract that caused a reduction in cell growth. Values are for mean ± SD for *n* = 4.

The cytotoxicity profile of the extracts of *D. kilimandscharicus* was determined by assessing for the cell viability of RAW 264.1 cells using the resazurin assay. The ethanol and ethyl acetate extracts were investigated as they had shown to be the most potent extract in reducing Jurkat-T cell viability. The results (Figure 4) demonstrated that the ethyl acetate extract had an IC_50_ of 428 *μ*g/mL and that of ethanol was 422 *μ*g/mL, and these values are considered nontoxic. The American National Cancer Institute guidelines set the limit of activity for crude extracts at a 50% inhibition (IC_50_) of proliferation of less than 30 *μ*g/mL after an exposure time of 72 hrs. The polar leaf extracts showed greater antiproliferative activity than nonpolar leaf extracts indicating that most of the compounds that may be responsible for the observed antiproliferative effect are more soluble in polar solvents.  4. The cytotoxicity screening of plants is useful that it provides information on the safety levels at which plants may be used without causing harm.

### 3.4. Chemical Composition of *Dolichos kilimandscharicus* Leaf Extract

**Figure 5:**
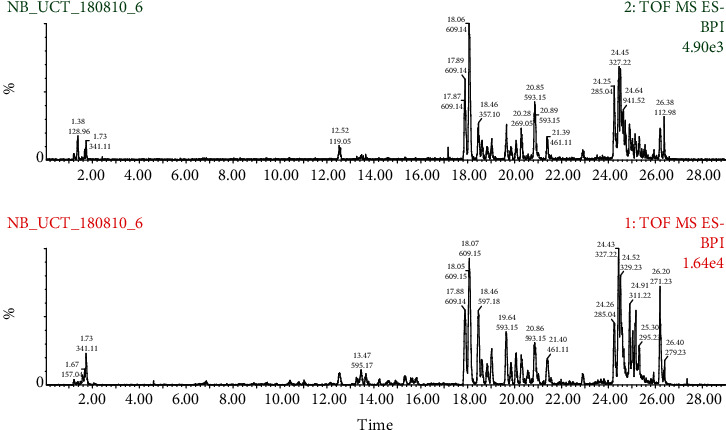
Typical chromatograms obtained from UPLC–MS analysis of crude leaf ethanol extract of *D. kilimandscharicus*. Total ion signal (TOF-MS) and retention times (in minutes) are indicated for the most intense peaks (the difference between the two detectors is 0.15 min).

Figure 5 illustrates a typical UPLC-MS/MS chromatogram of the ethanolic extract of *D. kilimandscharicus* leaves. The retention/acquisition times (*R*_*t*_) were reported in minutes. The corresponding mass spectra are shown in Table 1.

**Figure 6:**
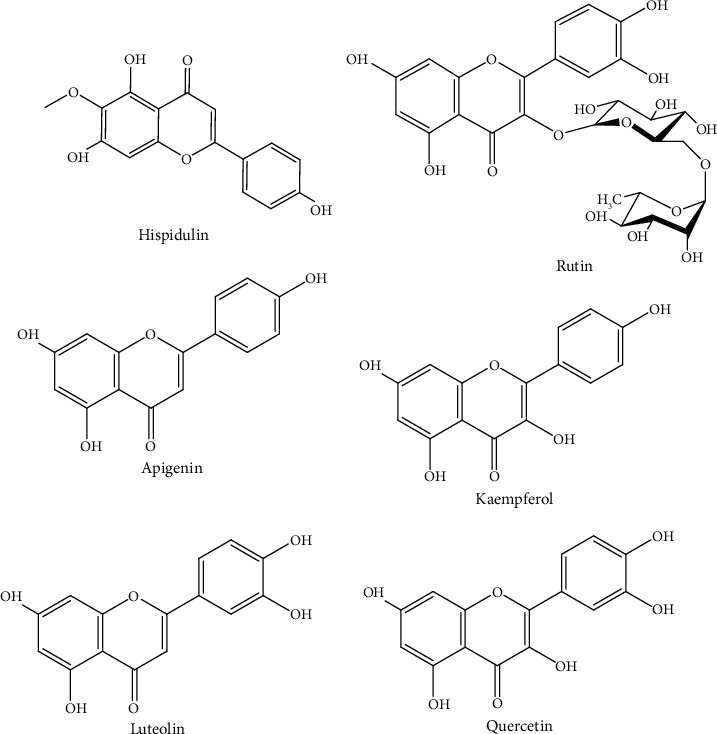
Chemical structures of compounds identified from UPLC–MS analysis of crude leaf ethanol extract. The compounds are probably responsible for the aforementioned therapeutic benefits.

The presence of these phytochemicals indicates the potential of *D. kilimandscharicus* leaves as a source of proliferation inhibitors. Our findings identified the most prominent antiproliferative compounds as rutin, quercetin, luteolin, apigenin, hispidulin, kaempferol derivatives, as well as caffeoylquinic acid whose structures are shown in Figure 6.

UPLC-MS analysis of crude extracts led to the identification of 23 components from the ethanol extract with precisely rutin, quercetin, luteolin, apigenin, hispidulin, kaempferol derivatives, as well as caffeoylquinic acid being the most prominent antiproliferative compounds. A number of studies have shown that rutin inhibits the proliferation of colon cancer, cervical, and human neuroblastoma by triggering apoptosis in these cells [23, 24]. Quercetin has been reported to possess antiproliferative effects on a wide range of cancer cells including colon carcinoma CT-26 cells, prostate adenocarcinoma LNCaP cells, human prostate PC3 cells, pheochromocytoma PC12 cells, estrogen receptor-positive breast cancer MCF-7 cells, acute lymphoblastic leukaemia MOLT-4 T-cells, human myeloma U266B1 cells, human lymphoid Raji cells, and ovarian cancer CHO cells [13, 25]. Luteolin exhibits a variety of inhibitory activities against various cancers such as lung, breast, glioblastoma, prostate, colon, and pancreatic cancers [24].

Apigenin from previous studies has been shown to potentially be used as a dietary supplement as well as cancer treatment agent [26]. In a study by Imran et al. [24], it was shown that hispidulin can act potentially in the control tumour progression and angiogenesis. Kaempferol and its glycoside derivatives can be found vastly in nature as well as showing antiproliferation effect on the human hepatoma cell line HepG2, mouse colon cancer cell line CT26, and mouse melanoma cell line B16F1 [27]. The activity of compounds against microorganisms was shown to depend largely on the structure of the compound [28]. The compound 5-O-caffeoylquinic acid has also been identified from the methanolic extracts of *Cydonia oblonga* Miller and has been shown to possess antiproliferative effects on human kidney and colon cancer cells [29]. However, the highly complex chemical compositions of the leaf extracts make it difficult to assign the growth-inhibitory effect to one particular compound present in *D. kilimandscharicus* unless the pure compounds are isolated and their structure elucidated.

The study of the constituent phytochemicals in medicinal herbs is pivotal to reveal the potential candidates in each medicinal herb that would likely explain their therapeutic effects. The study showed significant antiproliferative effect of the ethanol and ethyl acetate leaf extracts that has stimulated us to carry out ongoing work to determine the active compounds, that may prove to be valuable as lead compounds in their ability to kill Jurkat-T cells but exerting a marginal cytotoxic effect on normal cells. No data relating ?tex id="A80" author-cmt="Text corrected to make grammatical sense"?> to the components with antiproliferative effects of the two active leaf extracts have been reported and as such further phytochemical work on the isolation and identification of active structures of these extracts is required.

## 4. Conclusions

It can be concluded that the most active compounds of the plant may be in the ethanol and ethyl acetate extracts of D. kilimandscharicus. The ethanol and ethyl acetate extracts*D. kilimandscharicus* induced apoptosis in Jurkat-T cells and can, thus, be considered for further studies to identify compounds with potential anticancer activity. Furthermore, these extracts were found not to be cytotoxic against the RAW 264.7 normal cell line. UPLC-MS analysis of the leaf extracts led to the identification of 23 compounds from the ethanol extract, and these may be responsible for the observed antiproliferative effects. Rutin, quercetin, luteolin, apigenin, hispidulin, kaempferol derivatives, as well as caffeoylquinic acid are some of the compounds identified in the leaf extracts of D. kilimandscharicus and may be responsible for the observed effects.

## Figures and Tables

**Table 1:** Characterisation of tentatively identified compounds in the ethanol extract of *D. kilimandscharicus* by UPLC-MS.

Peak #	Retention time (min)	[M-H]^−^	Proposed formula	Error (ppm)	Proposed compounds
1	18.06	609.1448	C_27_H_30_O_6_	-1.3	Rutin
2	18.06	301.0320	C_15_H_10_O_7_	-1.0	Quercetin
3	18.45	285.0388	C_15_H_10_O_6_	-0.2	Luteolin
4	18.45	357.0973	C_19_H_17_O_7_	-3.9	2-[4-[2-(3,4-Dihydro-2H-1,5-benzodioxepin-7-yl)acetyl]-3-hydroxyphenoxy]acetate
6	18.45	579.1386	C_26_H_28_O_15_	0.2	Luteolin-7-O-glucosyl-3-O-arabinoside
7	18.45	447.0943	C_21_H_20_O_11_	1.6	Kaempferol 3-O-glucoside or luteolin hexoside
8	18.45	269.0440	C_15_H_10_O_5_	-3.7	Apigenin
9	20.85	299.0555	C_15_H_10_O_6_	-0.3	6-demethoxy-4′-O-methylcapillarisin
10	20.85	593.1525	C_27_H_30_O_15_	1.7	Kaempferol 3-O-rutinoside
11	20.85	623.1633	C_29_H_22_O_16_	1.9	Isorhamnetin 3-O-rutinoside
12	20.85	329.0650	C_17_H_14_O_7_	-3.3	Hispidulin
13	20.85	431.0978	C_21_H_22_O_10_	0.0	Apigenin 3-O-glucoside
14	21.39	461.1075	C_22_H_22_O_11_	-2.0	Methylkaempferol-hexose
15	21.39	283.0234	C_15_H_7_O_6_	-3.2	3,8-Dihydroxy-methyl anthraquinone carboxylic acid
16	21.39	593.1448	C_34_H_25_O_10_	-1.0	[3-[2-[2-(3,4-Dihydroxyphenyl)-3,5,7-trihydroxy-3,4-dihydro-2H-chromen-8-yl]ethenyl]-7-(4-hydroxyphenyl)-2,8-dioxatricyclo[7.3.1.05,13]trideca-1(12),3,5(13),6,9-pentaen-11-ylidene]oxidanium
17	21.39	255.0289	C_14_H_7_O_5_	-1.6	9-(18O)(18O)Oxidanylcarbonyl-6-oxoxanthen-3-olate
18	24.51	327.2162	C_18_H_10_O_6_	3.5	5-(7-Hydroxy-4-oxochromen-3-yl)-1-benzofuran-2-carboxylic acid
19	24.51	353.1014	C_16_H_18_O_9_	12.5	Caffeoylquinic acid
20	24.51	941.5145	C_66_H_99_O_5_	32.5	1-Palmityl-2-cholesterylcarbonoyl-3-trityl glycerol
21	24.51	353.1014	C_20_H_17_O_6_	12.5	3-(2,4-Dihydroxyphenyl)-5,7-dihydroxy-6-(3-methyl-2-butenyl)-4H-1-benzopyran-4-one
22	26.38	271.2268	C_16_H_31_O_3_	1.5	16-Hydroxypalmitate
23	26.38	529.2180	C_39_H_29_O_2_	25.5	4-[5-(2,6-Diphenylpyran-4-ylidene) penta-1,3-dienyl]-2,6-diphenylpyrylium

## Data Availability

The data used to support the findings of this study are available from the corresponding author upon request.
